# Supplementary material for: Dissipation Mechanisms and Superlubricity in Solid Lubrication by Wet-Transferred Solution-Processed Graphene Flakes: Implications for Micro Electromechanical Devices
Source: ACS Appl Nano Mater. 2023 Jun 15;6(13):11443–54. doi: 10.1021/acsanm.3c01477 (PMC10352959; doi:10.1021/acsanm.3c01477)
Supplement: Supplementary file 1 — an3c01477_si_001.pdf [file an3c01477_si_001.pdf]

Dissipation Mechanisms and Superlubricity in Solid Lubrication by Wet-Transferred Solution-Processed Graphene Flakes: Implications for Micro Electromechanical Devices

Renato Buzio*[1], Andrea Gerbi[1], Cristina Bernini[1], Luca Repetto[2], Andrea Silva[3,4] and Andrea Vanossi[3,4]

[1] CNR-SPIN, C.so F.M. Perrone 24, 16152 Genova, Italy
[2] Dipartimento di Fisica, Università degli Studi di Genova, Via Dodecaneso 33, 16146 Genova, Italy
[3] CNR-IOM Consiglio Nazionale delle Ricerche - Istituto Officina dei Materiali, c/o SISSA, Via Bonomea 265, 34136 Trieste, Italy
[4] International School for Advanced Studies (SISSA), Via Bonomea 265, 34136 Trieste, Italy

*Corresponding Author: Renato Buzio - Email: renato.buzio@spin.cnr.it

Supplementary Information

S1. Preparation of colloidal probes and force calibration

We assembled colloidal probes by gluing silica spheres of $25.24\mu m$ mean nominal diameter (MicroParticles GmbH SiO_2-R-SC93; standard deviation $0.75\mu m$) to rectangular Si cantilevers with molten Shell Epikote resin (heating temperature $\sim 140°C$), using a custom-built micromanipulation stage coupled with an optical microscope.[1] The nominal elastic constant of each cantilever (MikroMasch HQ:NSC35/tipless, NSC12/tipless) was measured by Sader's method[2] and was in the range $k_C \sim 3.1 - 4.4 N/m$.

The applied normal force F_N was estimated as $F_N = \alpha_N \Delta U_N$, where ΔU_N is the variation of the photodiode current induced by the cantilever vertical deflection and the normal force calibration factor $\alpha_N = k_N^* s_N$. Here, k_N^* is the effective spring constant of the colloidal probe[3] and s_N is the vertical deflection sensitivity of the AFM optical lever system. The lateral force calibration factor α_L, required to estimate the lateral force $F_L = \alpha_L \Delta U_L$, was determined by means of a diamagnetic levitation spring system.[4]

For calibration of normal forces, the sensitivity s_N (in nm/nA) was obtained from the slope of normal deflection *vs* displacement curves acquired over a rigid Si wafer. To obtain the effective spring constant of the colloidal probe k_N^*, we first evaluated the normal spring constant k_C of the bare silicon cantilevers (*i.e.* before attaching the silica bead) using Sader's method.[2] Next, we estimated the spring constant $k_N = k_C (L_C/L_B)^3$, where L_B is the distance of the glued bead with respect to the base of the lever (Figure S1).

Finally, following Edwards *et al.*[3], the effective spring constant k_N^* of the colloidal probe was obtained as:

$$k_N^* \equiv k_N \frac{1}{\cos^2\theta} \frac{1}{\left(1 - \frac{3R}{2L_B} tan\theta\right)}$$

where $\theta \approx 20°$ is the cantilever tilt with respect to the sample surface, and $R = 12.6\mu m$ is the nominal bead radius. The normal force F_N was thus calculated as $F_N = \alpha_N \Delta U_N = k_N^* s_N \Delta U_N$.

The calibration of lateral force values F_L was accomplished by means of a diamagnetic levitation spring system of lateral stiffness ~82pN/nm (levitated mass $m^L \approx 44.0$ mg; $\omega_d(x_1) \approx 43.095$ rad/s and $\omega_d(x_2) \approx 44.267$ rad/s; $k_{11}(x_1) \approx 81.7$ pN/nm; see[4] for details and notation). Linear interpolation of plots of the lateral deflection signal ΔU_L (in nA) against the lateral spring

displacement x_1 (hence the spring force $k_{11}x_1$, in nN) provided the lateral force calibration factor α_L (in nN/nA). The lateral force F_L was thus calculated as $F_L = \alpha_L \Delta U_L$.

In the present study, $\alpha_N \sim 510 - 850 \mathrm{nN/nA}$ and $\alpha_L \sim 1.0 - 2.0 \mathrm{\mu N/nA}$, depending on the actual properties of the tipless cantilever and on the bead position along the long axis of the cantilever.

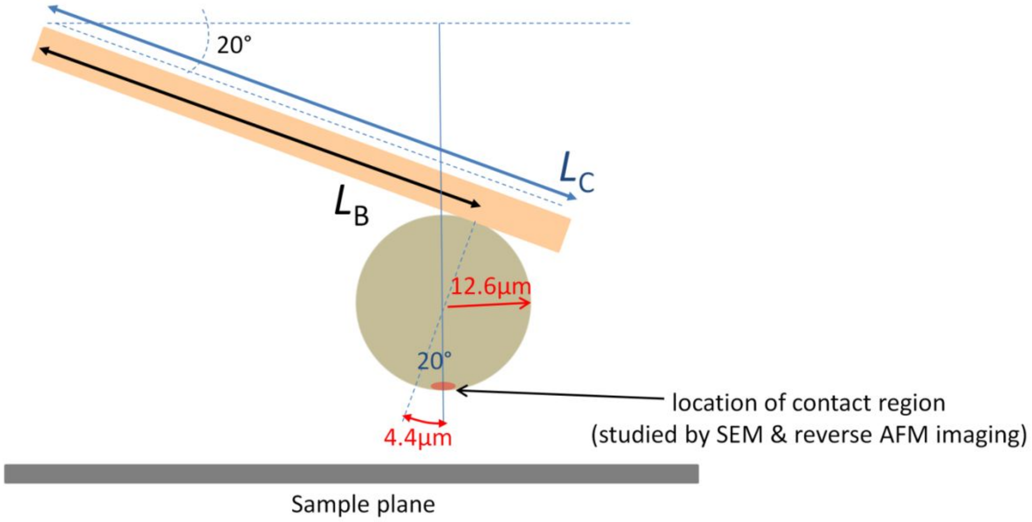

Figure S1. Side view of the colloidal probe during AFM experiments. The angle of 20°, formed by the cantilever's long axis with the plane of the sample surface, is imposed by the AFM head design. This angle is taken into account for the calibration of normal forces as well as for the exact location of the contact region in SEM imaging.

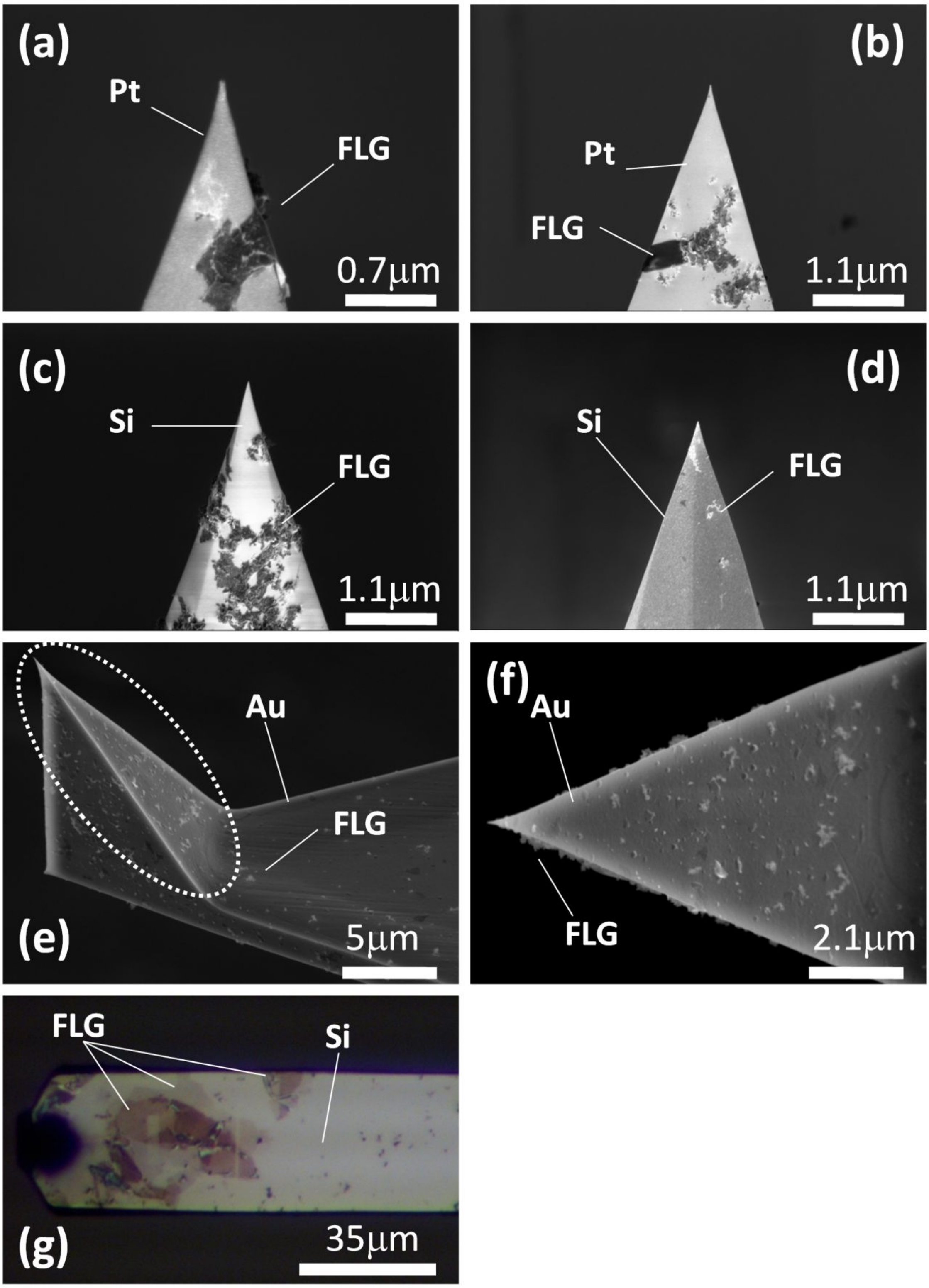

Figure S2. (a-f) SEM micrographs of commercial AFM probes dipped into Eau de Graphene solution. Despite variations of the preparation protocol and probes material there was no evidence for graphene-wrapped tips, albeit FLG flakes were clearly discerned over different regions of the probes' surface. (a),(b) Pt-coated probes (HQ:NSC35/Pt by MikroMasch), treated by oxygen plasma (30W, 30s, working pressure p_{O_2}~1.5×10^{-1}mbar) prior to immersion into graphene solution (dipping time 36 minutes). (c),(d) Si probe (HQ:CSC38/Al-BS by MikroMasch), treated by oxygen plasma (as in (a)-(b)) and dipped into graphene solution (dipping time 100 minutes). (e) Au-coated probe (custom OMCL-AC160TS by Olympus) dipped into graphene solution (dipping time 40 minutes). (f) Magnification of the dotted region in (e). (g) Optical micrograph of the tipped end of an AFM probe (upside tip on the left side) showing the

effective transfer of very large flakes from the liquid phase to the cantilever surface (oxygen plasma 30W, 30s, working pressure p_{O_2}~1.5×10^{-1}mbar; dipping time 28h). Among all the performed trials, this was the only case attesting deposition of large micrometric flakes from solution to an AFM probe.

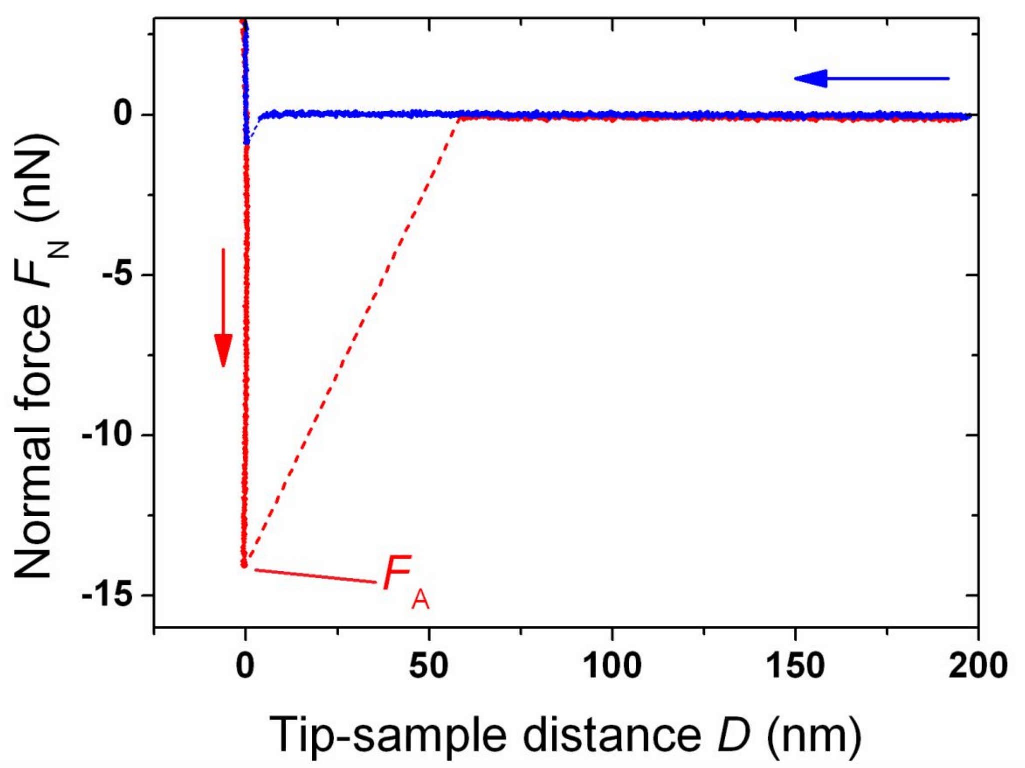

Figure S3. Normal force *vs* distance (F_N vs D) curve acquired on HOPG with the graphene-wrapped AFM probe of Fig. 3(a),(b) (see main text). The curve shows sharp snap-in-contact and snap-off-contact, and an adhesion force F_A $\cong -14$nN.

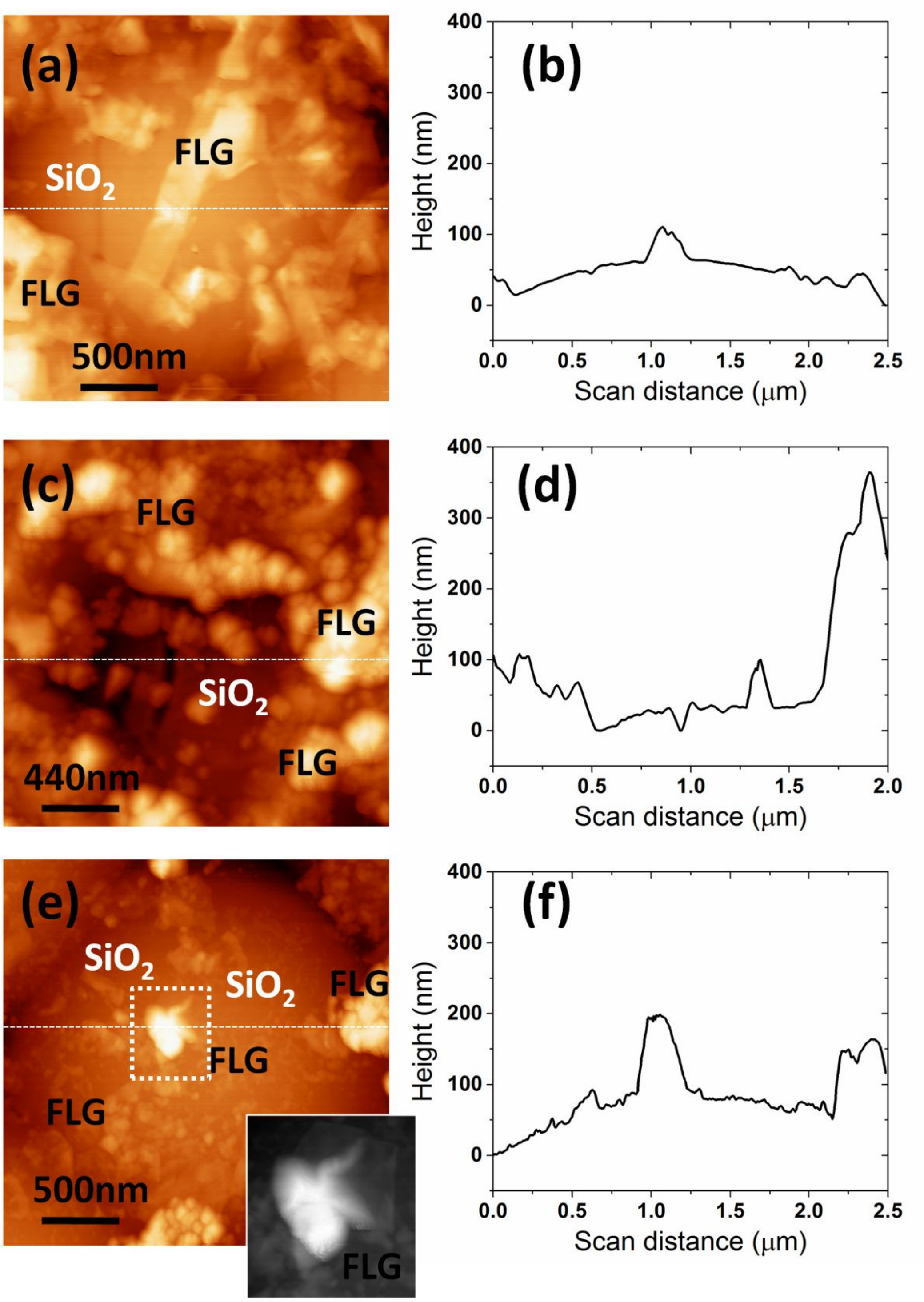

Figure S4. Reverse AFM morphologies (left column) and cross-section heights (along the dash lines; right column) for three silica beads coated by wet-transferred flakes. (a-b) They correspond to the colloidal bead of Figure 3c,d and Figure 4 in the main text. (c-d) They correspond to the colloidal bead named "Coated Probe 1" (see Figure 3e,f, Figure 5b and Figure 6 of the main text). (e-f) They correspond to the colloidal bead named "Coated Probe 2" (Figure 5c of the main text).

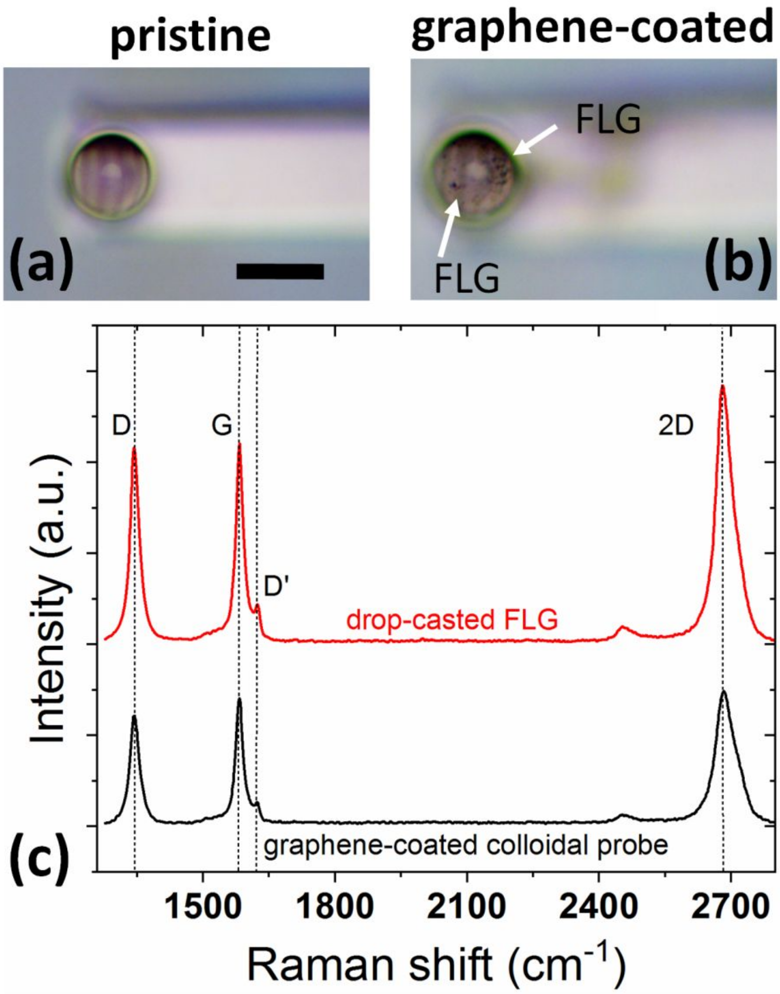

Figure S5. Optical micrographs of (a) pristine colloidal AFM probe and (b) the same probe after being coated by FLG flakes (scale bar in (a) is 27μm)). (c) Comparison of Raman spectra collected respectively on drop-casted FLG flakes (red curve) and at the surface of the graphene-coated probe (black curve).

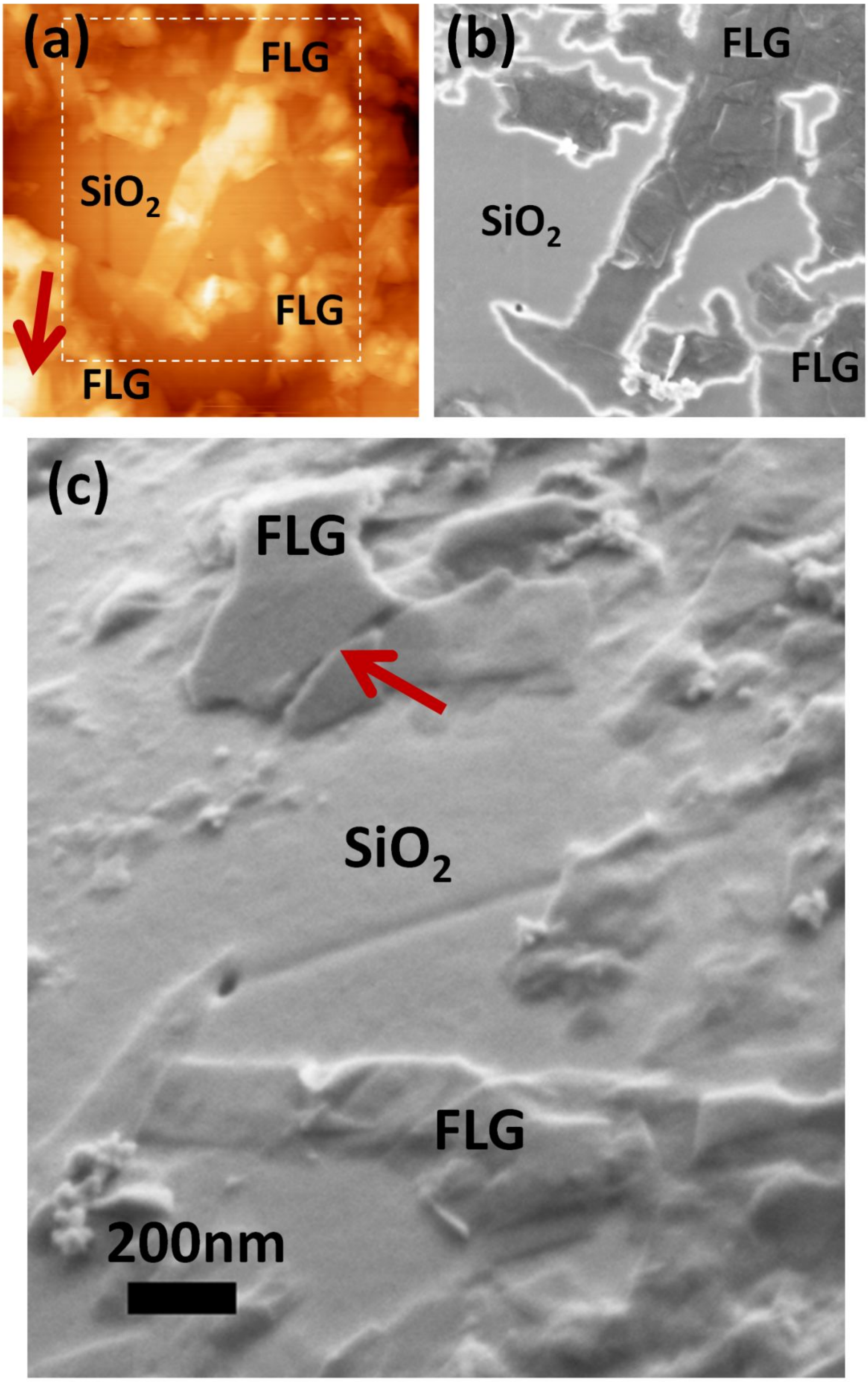

Figure S6. (a) AFM morphology acquired by reverse imaging nearby the contact region of the graphene-coated colloidal probe of Figure 3c,d and Figure 4i. (b),(c) SEM micrographs of the same region, from different viewpoints. The arrow highlights the location of the topographically-highest contact asperity.

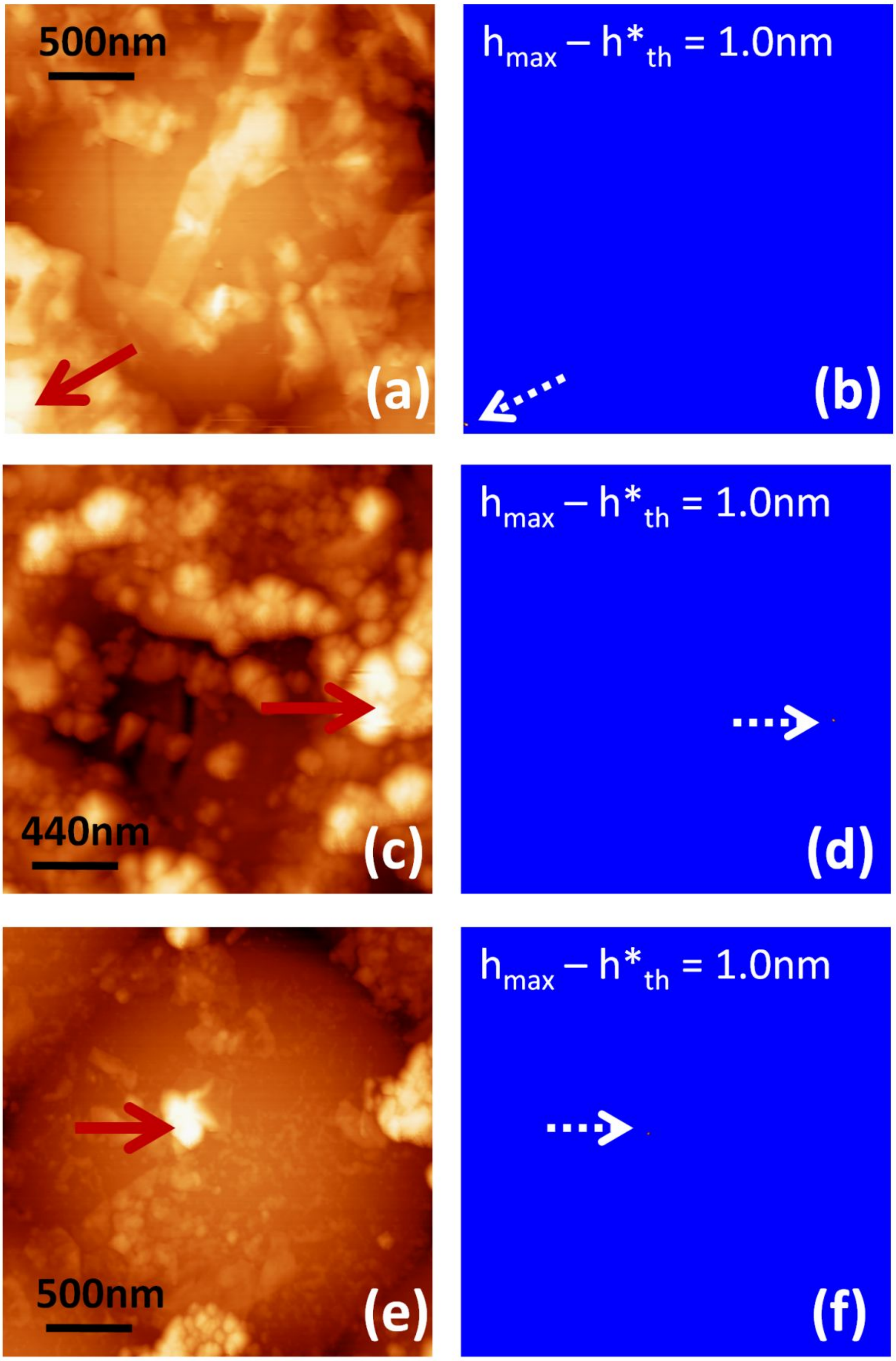

Figure S7. Estimation of the area of the topographically-highest nanoasperity for three graphene-coated colloidal probes. The area of the topographically-highest nanoasperity is roughly estimated by processing the topographies in (a),(c),(e) with the "flooding" option of the WSXM software. This method is based on setting all the image values below a given threshold value h_{th} (selected by the user) to a constant value. The resulting image resembles a picture of a flooded region of land (Figure S6, right column). Once the process is finished WSXM can calculate the area $A_{isl}(h_{th})$ of the "drought islands". The threshold value h_{th} was varied for each topography in small steps of $0.1 - 0.2$nm below the maximum surface height h_{max}, to find out the threshold h^*_{th} separating the regime of single-asperity contact (

$0 < h_{th} \leq h_{th}^*$, only one island in the "flooded topography") from a multi-asperity contact ($h_{th} > h_{th}^*$, two or more islands in the "flooded" topography). The area of the topographically-highest nanoasperity was estimated as $A_{isl}(h_{max} - h_{th}^*)$. We conventionally assumed that h_{th}^* values that are more than 1nm apart from h_{max} (i.e. $h_{max} - h_{th}^* > 1\text{nm}$) are unphysical. In fact the penetration depth of a nanoasperity into graphite is expected to be of only $\delta \sim 0.3$ - 0.6nm at the normal load $F_N = 100\text{nN}$ (from Hertzian contact theory $\delta = (F_N^2/E^2R)^{1/3}$ with the effective Young modulus $E \sim 30$ GPa and a tentative curvature radius $R = 50 - 300\text{nm}$ for the nanoasperity). In such specific situations the area of the topographically-highest nanoasperity was assumed to be $A_{isl}(h_{max}$ - 1nm). We obtained that A_{isl} is $\sim 2 \times 10^2$ nm^2 for all the beads. Arrows in the original AFM topographies (left column) and "flooded topographies" (right column) highlight the location of the single "drought island".

S8. Nonlinear force *vs* distance curves measured by means of the pristine colloidal probes

In a previous study[5] we discussed the origin of the adhesion F_A for pristine colloidal probes in contact with HOPG. We showed that the long-range van der Waals (vdW) force gives only a modest contribution to F_A. Assuming that the colloidal bead roughness limits the distance of closest approach to HOPG to about $\sim 1\text{nm}$, we estimated a vdW attraction of $\sim 209\text{nN}$ (see Equation A12 in the Supplementary Information of [5]); this is much smaller that the adhesion measured in experiments. On the other side, using the theoretical model by Farshchi-Tabrizi *et al.*,[6] we calculated that the capillary force of a water meniscus condensed around the bead-HOPG contact amounts to $\sim 2.6\mu\text{N}$ at the ambient relative humidity $\text{RH} = 60\%$ (see Equation A10 in the Supplementary Information of [5]). Therefore we concluded that the (total) estimated adhesion is dominated by the capillary force and amounts to $\sim 2.8\mu\text{N}$, which is comparable with F_A measured in experiments. Accordingly, the unloading branch of the force *vs* distance curves should mainly reflect the kinetics of rupture of the capillary bridge at the bead-HOPG contact.

We found nonlinear unloading curves for all the pristine probes in contact with HOPG, albeit the magnitude of the effect varied from probe to probe. Nonlinearity took place in the tensile region $F_N < 0$ a few hundreds of nm before the jump-off-contact. We believe that the nonlinearity derives from the superposition of distinct contributions.

Intrinsic contributions from cantilever tilt and contact mechanics. Qualitatively, the nonlinear behavior depicted in Figure 5(a) agrees with the picture that on retracting the probe from the surface a capillary neck is built, then it shrinks and at some point it breaks. In fact it is known that similar phenomena can deviate the unloading branch from linearity, and they can even transform the sharp jump-of-contact detachment into a smoother slide-off-contact event.[7] In our experimental set-up, the cantilever is tilted by 20° with respect to the sample plane so that the tip end of the lever typically displaces (over the sample surface) in the cantilever long-axis direction whenever the contact load F_N is varied through a Δz elongation/retraction of the AFM scanner. This implies that the contact spot between the colloidal bead and the substrate is not stationary in the sample plane but it slides horizontally forward/backward by a distance $|\Delta x| \approx tan20° \, |\Delta z|$ during both loading/unloading branches.[8] For a typical scanner displacement $|\Delta z| = 1\mu m$ one gets $|\Delta x| \approx 320$ nm. Therefore, both the horizontal translation of the contact spot (imposing a shear force) and the vertical increase of the probe-surface gap (imposing a tensile force) contribute in principle to the

kinetics of the capillary rupture, hence to the shape of the unloading curve nearby the jump-off-contact event. Another strictly related issue (certainly affecting the unloading branch) is the frictionally-driven torque of the colloidal probe. In fact, due to the horizontal translation of the contact spot during a force *vs* distance curve, longitudinal friction forces come into play producing a torque that opposes the normal bending moment.[9] In our experiments we often found – as an evidence of the sizeable role of the frictionally-driven torque – the manifestation of a peculiar hysteresis between the forward and backward branches of the force *vs* displacement curves (e.g. see Figure 1(b) in [9]).

Extrinsic contribution from the AFM Position-Sensitive-Photo-Detector (PSPD). A careful evaluation of the maximum tensile force $F_{\mathrm{N}}^{\mathrm{max}}$ (i.e. the maximum vertical deflection signal $\Delta U_{\mathrm{N}}^{\mathrm{max}}$) that can be measured for each colloidal probe by means of the PSPD shows that the value of the experimentally-measured adhesion $F_A \sim 2-3\mu\mathrm{N}$ was always very close to F_N^{max} ($F_A/F_N^{\mathrm{max}} \sim 0.8-0.9$). Under such working conditions, the PSPD behaves nonlinearly because its response depends on the finite size and shape of the laser spot reflected by the backside of the cantilever into the PSPD active area. An analytical expression for the PSPD response that takes into account the finite size effects of an ideally-round spot is:

$$\Delta U_N(\delta) = \Delta U_{\mathrm{N}}^{\mathrm{max}} \frac{2}{\pi}\left(\arcsin \frac{\beta\delta}{R_{spot}} + \frac{\beta\delta}{R_{spot}} \sqrt{1 - \frac{\beta^2\delta^2}{R_{spot}{}^2}} \right)$$

where R_{spot} is the laser spot radius, δ is the cantilever deflection and β is the optical level amplification factor. When the unloading branch is measured on an ideally-rigid and adhesive substrate $\delta = \Delta z$, and one gets the PSPD linear scaling $\Delta U_N(\Delta z) \propto \Delta z$ for $\Delta z \ll R_{spot}/\beta$ whereas $\Delta U_N(\Delta z) \rightarrow \Delta U_{\mathrm{N}}^{\mathrm{max}}$ for $\Delta z \rightarrow R_{spot}/\beta$. A solution to mitigate the extrinsic PSPD contribution is to use elastically stiffer cantilevers in order to reduce their deflection signal ΔU_N at the jump-of-contact, and thus make the optical level system always operate in the PSPD linear regime. However we avoided to use stiffer cantilevers, as this would have considerably decreased the sensitivity to lateral forces required to explore the superlubric response of the graphene-coated probes. In practice, the extrinsic contribution of the PSPD implies that the measured jump-of-contact amplitude underestimates the true adhesion force F_A experienced by the pristine probes on HOPG.

We note that the above discussion has no impact on the phenomenon of the 'adhesion breakdown' depicted in Figure 4(k) and Figure 5(a),(b), as this holds regardless of the intrinsic/extrinsic nature of the nonlinearity. We also underline that AFM force spectroscopy experiments involving the

graphene-coated probes are not affected by the PSPD nonlinearity (as the maximum contact force F_N amounts to $\sim 700 nN \ll |F_N^{\max}|$, see Figure 6(d)).

Since the nonlinearity of the unloading branch reflects multiple contributions besides contact mechanics effects, we avoided to transform experimental $F_N\ vs\ z$ curves into $F_N\ vs\ D$ curves for the case of the pristine colloidal probes.

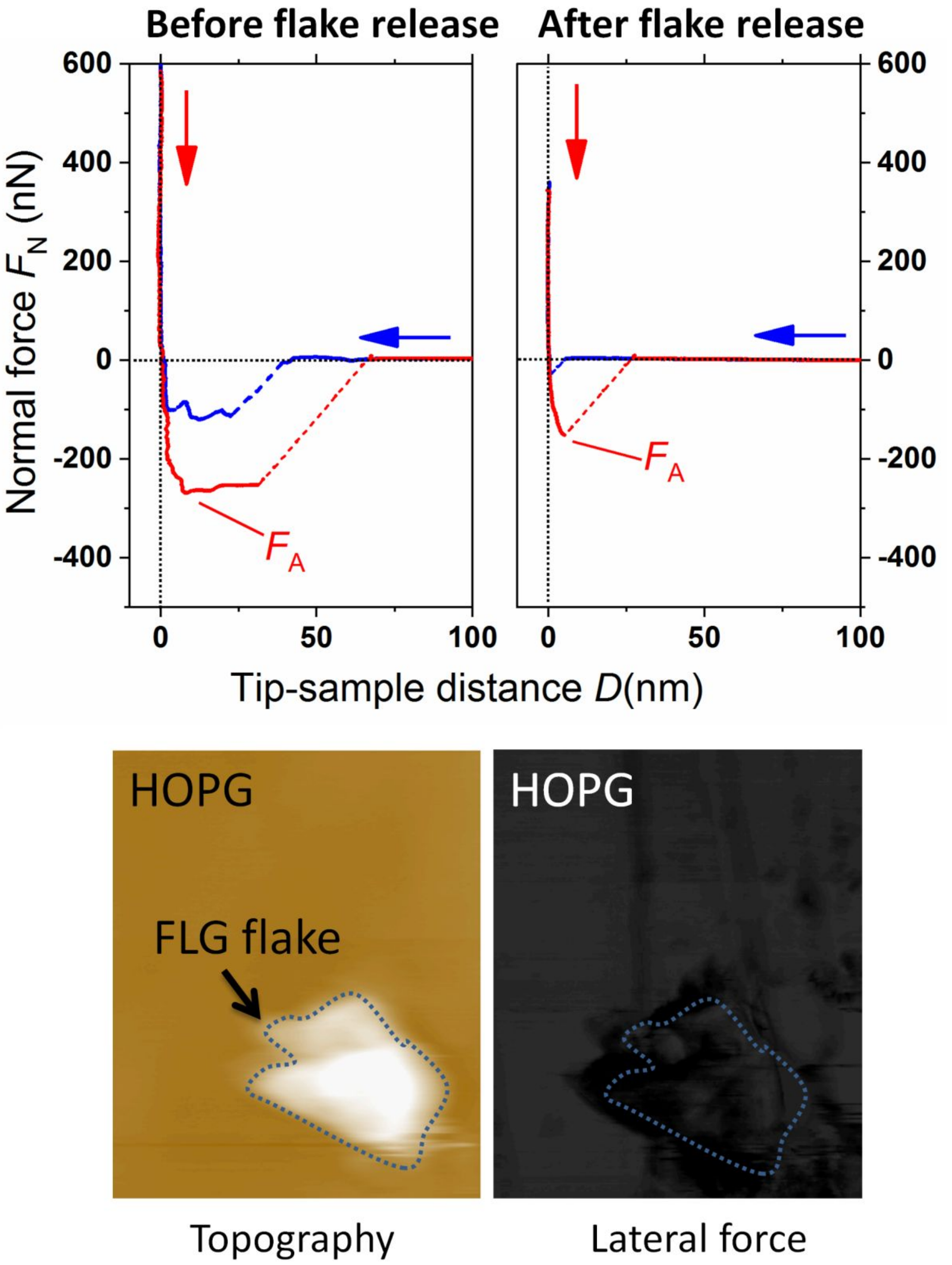

Figure S9. Variation of the long-ranged behavior of two spectroscopic curves (top) acquired with the very same graphene-coated colloidal probe on HOPG. Variation is triggered by the release of a loosely attached FLG flake from the coated probe to HOPG (occurred while sliding on HOPG in between the two spectroscopic experiments).

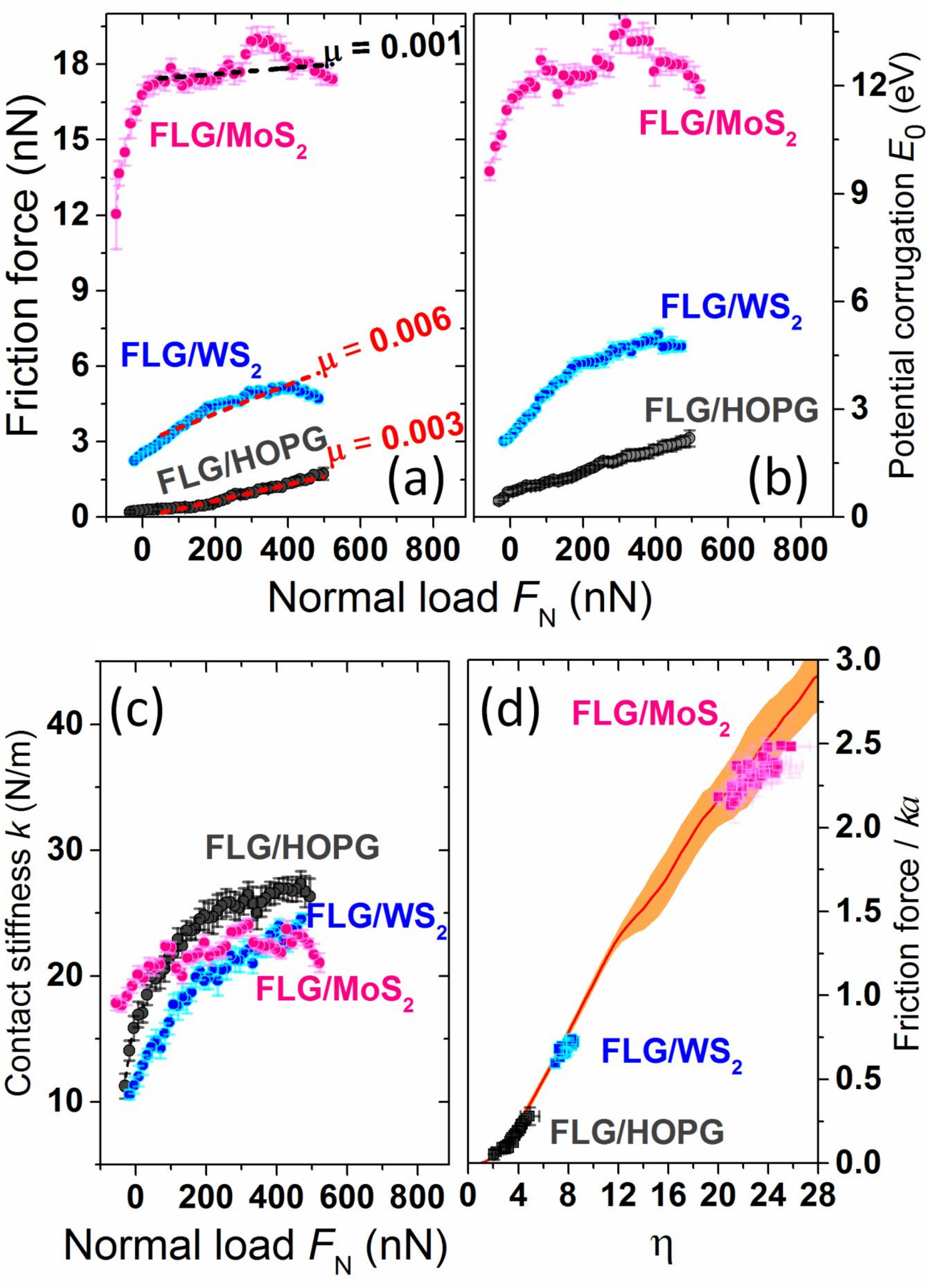

Figure S10. (a) Representative F_f vs F_N characteristics measured by means of the colloidal probe shown in Figure S3a and Figure S5 (see also main text Figure 3c,d) for the three layered sliding junctions FLG/HOPG, FLG/WS_2 and FLG/MoS_2 respectively ($v = 33$nm/s). (b)-(c) Load-dependent variation of the interfacial parameters E_0 and k. (d) Comparison of experimental data with the PT model. Data are represented by square symbols as in Figure 6i.

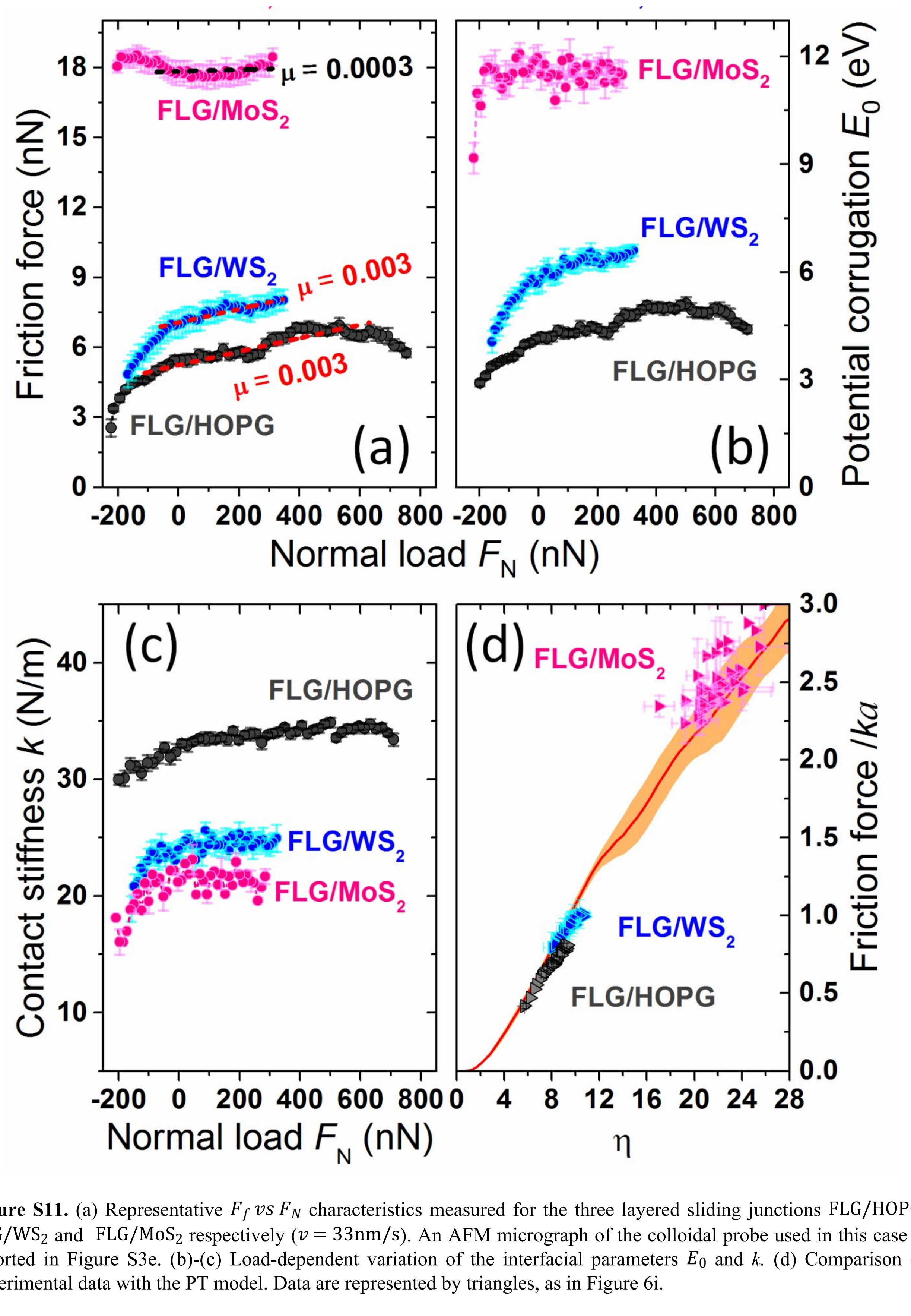

Figure S11. (a) Representative F_f vs F_N characteristics measured for the three layered sliding junctions FLG/HOPG, FLG/WS_2 and FLG/MoS_2 respectively ($v = 33\mathrm{nm/s}$). An AFM micrograph of the colloidal probe used in this case is reported in Figure S3e. (b)-(c) Load-dependent variation of the interfacial parameters E_0 and k. (d) Comparison of experimental data with the PT model. Data are represented by triangles, as in Figure 6i.

S12. Role of damping in stick-slip dynamics

To better support our claim that the downward flexion of $F_f^* \equiv F_f/ak \ vs \ \eta$ curves (Figure 6i) is probably due to the underdamped frictional dynamics, we carried out simulations at varying Langevin damping parameter γ. To isolate the effect of damping and make results as clear as possible, we carried out the simulations at zero temperature T=0K. The lateral spring constant was fixed at $k = 10\text{N/m}$. Figure S12 reports the normalized friction force as a function of the Tomlinson parameter η, for various values of the damping γ, along with the theoretical limit by Gnecco et al.[10] in the zero-temperature single-slip overdamped case. For large γ, the simulated curve follows the theoretical prediction (e.g. $\gamma = 0.137\text{ns}^{-1}$, purple triangles in Figure S12(a)). As γ decreases, the curve develops the downward trend discussed in the main text, as expected.

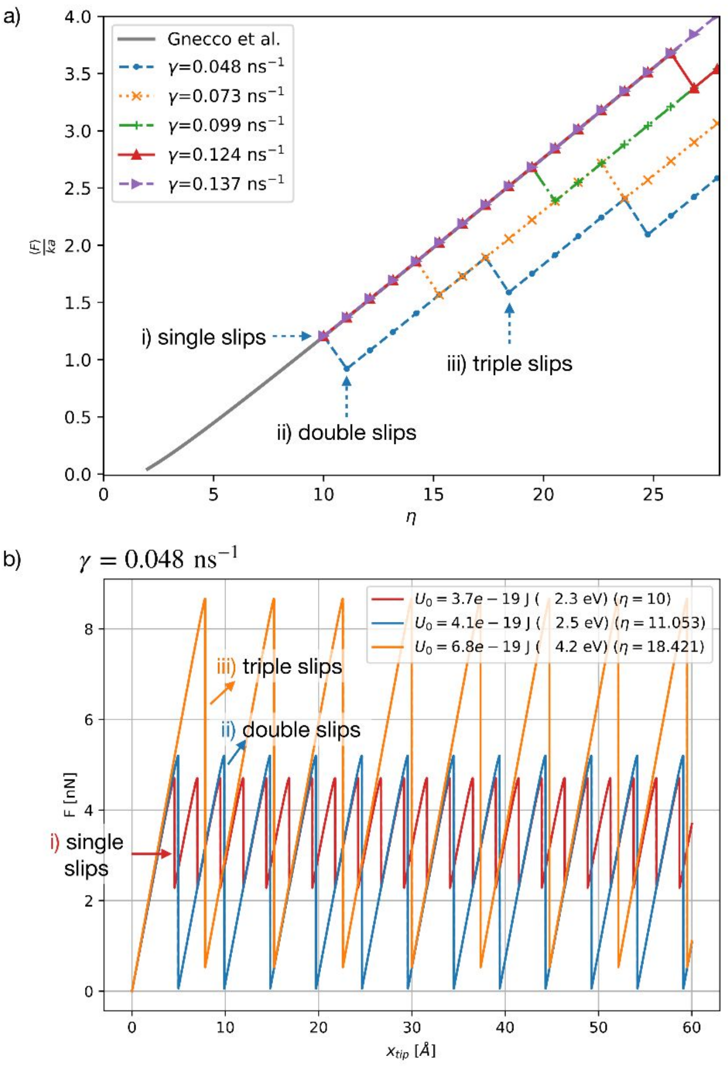

Figure S12. (a) Normalized friction force F/ak as a function η, for the values of the damping γ reported in the legend. The solid gray line shows the analytical result from Gnecco *et al.*[10] (b) Force traces as a function of tip position for the three values of U_0 (thus η) reported in the legend. The labels mark the traces corresponding to single, double, and triple slips. The traces where obtained at $\gamma = 0.048\text{ns}^{-1}$.

Moreover, these underdamped zero-temperature curves show sharp steps as a function of η, marked in Figure S12(a) for $\gamma = 0.048\text{ns}^{-1}$. These steps correspond to the transition from single to double, triple, etc. slips. This is clearly shown in Figure S12(b) for $\gamma = 0.048\text{ns}^{-1}$: the force traces at different substrate potential U_0 (hence η) evolve from single to multiple stick-slips. Hence, for smaller Langevin dissipation, inertia allows the transition from single to multiple stick-slip to occur at a smaller value of η, while for very large dissipation, only single slips are allowed as predicted by Gnecco *et al.*[10]. This mechanism underpins the downward trend as a function of η observed in experimental data, validating our assumption of underpinned dynamics.

References

(1) Buzio, R.; Valbusa, U. Interfacial Stiffness and Adhesion of Randomly Rough Contacts Probed by Elastomer Colloidal AFM Probes. *J. Phys. Condens. Matter* **2008**, *20* (35), 354014. https://doi.org/10.1088/0953-8984/20/35/354014.

(2) Green, C. P.; Lioe, H.; Cleveland, J. P.; Proksch, R.; Mulvaney, P.; Sader, J. E. Normal and Torsional Spring Constants of Atomic Force Microscope Cantilevers. *Rev. Sci. Instrum.* **2004**, *75* (6), 1988–1996. https://doi.org/10.1063/1.1753100.

(3) Edwards, S. A.; Ducker, W. A.; Sader, J. E. Influence of Atomic Force Microscope Cantilever Tilt and Induced Torque on Force Measurements. *J. Appl. Phys.* **2008**, *103* (6), 64513. https://doi.org/10.1063/1.2885734.

(4) Li, Q.; Kim, K.-S.; Rydberg, A. Lateral Force Calibration of an Atomic Force Microscope with a Diamagnetic Levitation Spring System. *Rev. Sci. Instrum.* **2006**, *77* (6), 65105. https://doi.org/10.1063/1.2209953.

(5) Buzio, R.; Gerbi, A.; Bernini, C.; Repetto, L.; Vanossi, A. Graphite Superlubricity Enabled by Triboinduced Nanocontacts. *Carbon* **2021**, *184*, 875–890. https://doi.org/10.1016/j.carbon.2021.08.071.

(6) Farshchi-Tabrizi, M.; Kappl, M.; Cheng, Y. J.; Gutmann, J.; Butt, H. J. On the Adhesion between Fine Particles and Nanocontacts: An Atomic Force Microscope Study. *Langmuir* **2006**, *22* (5), 2171–2184. https://doi.org/10.1021/la052760z.

(7) Cappella, B. *Mechanical Properties of Polymers Measured through AFM Force-Distance Curves*; 2016.

(8) Cannara, R. J.; Brukman, M. J.; Carpick, R. W. Cantilever Tilt Compensation for Variable-Load Atomic Force Microscopy. *Rev. Sci. Instrum.* **2005**, *76* (5). https://doi.org/10.1063/1.1896624.

(9) Chung, K. H.; Shaw, G. A.; Pratt, J. R. Accurate Noncontact Calibration of Colloidal Probe Sensitivities in Atomic Force Microscopy. *Rev. Sci. Instrum.* **2009**, *80* (6). https://doi.org/10.1063/1.3152335.

(10) Gnecco, E.; Roth, R.; Baratoff, A. Analytical Expressions for the Kinetic Friction in the Prandtl-Tomlinson Model. *Phys. Rev. B* **2012**, *86* (3), 35443. https://doi.org/10.1103/PhysRevB.86.035443.
